# Retraction: LncRNA TUG1 promotes the progression of colorectal cancer via the miR-138-5p/ZEB2 axis

**DOI:** 10.1042/BSR20201025_RET

**Published:** 2026-09-09

**Authors:** Zhenkun Yan, Miaomiao Bi, Qiyu Zhang, Yumei Song, Sen Hong

**Affiliations:** 1 Department of Endoscopy Center, China-Japan Union Hospital of Jilin University, Changchun 130022, Jilin Province, P.R. China; 2 Department of Ophthalmology, China-Japan Union Hospital of Jilin University, Changchun, Jilin 130033, P.R. China; 3 Department of Radiology, Jilin Oil Field Hospital, SongYuan, Jilin 138000, P.R. China; 4 Department of Thoracic Oncology, Tumor Hospital of Jilin Province, Jilin 130000, P.R. China; 5 Department of Colorectal and Anal Surgery, The First Hospital of Jilin University, Changchun 130021, Jilin Province, P.R. China

**Keywords:** biological function, CRC, miR-138-5p, TUG1, ZEB2

Following the Expression of Concern issued for this article, this article is now being retracted from *Bioscience Reports* at the request of the Editor-in-Chief and the Editorial Board. This follows the receipt of a notification from a reader, alerting the Editorial Board to an instance of high similarity between the Figure 1A and 1D panels, along with concerns over the appearance and style of the western blots, which share similar characteristics to a large group of unrelated articles.

The authors were contacted regarding the concerns raised and provided data from subsequent repeated experiments; however, no original data was able to be provided.

Given the extent of the issues raised, the Editorial Board stands by the decision to retract the article. The authors agree to the retraction.

